# An Investigation into the Registration of Unmanned Surface Vehicle (USV)–Unmanned Aerial Vehicle (UAV) and UAV–UAV Point Cloud Models

**DOI:** 10.3390/s25226992

**Published:** 2025-11-15

**Authors:** Yu-Shen Hsiao, Yu-Hsuan Cho, Yu-Sian Yan

**Affiliations:** Department of Soil and Water Conservation, National Chung Hsing University, Taichung 402, Taiwan

**Keywords:** USV, UAV, coordinate transformation, point cloud model

## Abstract

This study explores the integration of point cloud data obtained from unmanned aerial vehicles (UAVs) and unmanned surface vehicles (USVs) to address limitations in photogrammetry and to create comprehensive models of aquatic environments. The UAV platform (AUTEL EVO II) employs structure-from-motion (SfM) photogrammetry using optical imagery, while the USV (equipped with a NORBIT iWBMS multibeam sonar system) collects underwater bathymetric data. UAVs commonly face constraints in battery life and image-processing capacity, making it necessary to merge smaller UAV point clouds into larger, more complete models. The USV-derived bathymetric data are integrated with UAV-derived surface data to construct unified terrain models that include both above-water and underwater features. This study evaluates three coordinate transformation (CT) methods—4-parameter, 6-parameter, and 7-parameter—across three study areas in Taiwan to assess their effectiveness in registering USV–UAV and UAV–UAV point clouds. For USV–UAV integration, all CT methods improved alignment accuracy compared with results without CT, achieving decimeter-level precision. For UAV–UAV integrations, the 7-parameter method provided the best accuracy, especially in areas with low terrain roughness such as rooftops and pavements, while improvements were less pronounced in areas with high roughness such as tree canopies. These findings demonstrate that the 7-parameter CT method offers an effective and straightforward approach for accurate point cloud integration from different platforms and sensors.

## 1. Introduction

With the rapid development of terrestrial laser scanning (TLS), unmanned aerial vehicle (UAV) photogrammetry, and UAV light detection and ranging (LiDAR), point cloud models have become essential tools for describing and analyzing object shapes [1]. Point cloud models are now widely utilized across various fields, including environmental monitoring, autonomous navigation, archeological site recording, and urban modeling. In many of these applications, integrating point cloud data from different scanners or sensors is essential to produce complete and accurate 3D models. This work is crucial because of the variations in coordinate systems, resolutions, and measurement accuracies. Proper registration or fusion harmonizes these discrepancies by aligning data to a consistent reference frame, ensuring precise spatial relationships and comprehensive 3D models. This process mitigates the errors and inconsistencies arising from diverse sensor characteristics, enhancing the overall quality and usability of the fused point cloud models.

In the past 10 years, research on the registration of point cloud models has commonly included studies on TLS-TLSs and TLS-UAVs. For example, ref. [2] presented a practical framework for the integration of UAV-based photogrammetry and TLS in open-pit mine areas. The results show that TLS-derived point clouds can be used as ground control points (GCPs) in mountainous areas or high-risk environments where it is difficult to conduct a global navigation satellite system (GNSS) survey. The framework achieved decimeter-level accuracy for the generated digital surface model (DSM) and digital orthophoto map. Ref. [3] proposed an efficient registration method based on a genetic algorithm for the automatic alignment of two terrestrial laser scanning (TLS) point clouds (TLS–TLS) and the alignment between TLS and unmanned aerial vehicle (UAV)–LiDAR point clouds (TLS–UAV LiDAR). The experimental results indicate that the root-mean-square error (RMSE) of the TLS–TLS registration is 3–5 mm, and that of the TLS–UAV LiDAR registration is 2–4 cm. Ref. [4] discussed creating virtual environments from 3D point-cloud data suitable for immersive and interactive virtual reality. Both TLS (LiDAR-based) and UAV photogrammetric point clouds were utilized. The UAV point clouds were generated using optical imagery processed through structure-from-motion (SfM) photogrammetry. These datasets were merged using a custom algorithm that identifies data gaps in the TLS dataset and fills them with data from the UAV photogrammetric model. The result demonstrated an RMSE accuracy of approximately 5 cm. Ref. [5] designed a method for the global refinement of TLS point clouds on the basis of plane-to-plane correspondences. The experimental results show that the proposed plane-based matching algorithm efficiently finds plane correspondences in partial overlapping scans, providing approximate values for global registration, and indicating that an accuracy better than 8 cm can be achieved. Ref. [6] extracted key points with stronger expression, expanded the use of multi-view convolutional neural networks (MVCNNs) in point cloud registration, and adopted a graphics processing unit (GPU) to accelerate the matrix calculation. The experimental results demonstrated that this method significantly improves registration efficiency while maintaining an RMSE accuracy of 3 to 4 cm. Ref. [7] used a feature-level point cloud fusion method to process point cloud data from TLS and UAV LiDAR. The results show that the tally can be achieved quickly and accurately via feature-level fusion of the two point cloud datasets. Ref. [8] established a high-precision, complete, and realistic bridge model by integrating UAV image data and TLS point cloud data. The integration of UAV image point clouds with TLS point clouds is achieved via the iterative closest point (ICP) algorithm, followed by the creation of a triangulated irregular network (TIN) model and texture mapping via Context Capture 2023 software. The geometric accuracies of the integrated model in the X, Y, and Z directions are 1.2 cm, 0.8 cm, and 0.9 cm, respectively. Other related research on point cloud registration or fusion from TLS-TLS or TLS-UAV over the past ten years has been included [9,10,11,12,13,14,15,16,17,18,19,20,21,22,23].

According to the references, the fusion of TLS–TLS point clouds currently achieves millimeter-level accuracy, while the fusion of TLS–UAV point clouds attains centimeter-level accuracy. Compared with studies on TLS–TLS and UAV–TLS data integration, research specifically addressing the precise registration between UAV-derived point clouds from different flight missions or sensors (e.g., photogrammetry and LiDAR) remains limited. Ref. [24] presented a novel procedure for fine registration of UAV-derived point clouds by aligning planar roof features. The experimental results demonstrated an average error of 9 cm from the reference distances.

The integration of UAV-UAV point clouds is critical for photogrammetry. Affordable UAVs used for this purpose are often constrained by limited battery capacity and software limitations in processing large volumes of imagery, which makes it challenging to generate comprehensive point cloud models. This challenge can be addressed by merging smaller, individual UAV point cloud models into larger, more complete models. In addition, while the previous studies on TLS-TLS, UAV-TLS, and UAV-UAV point cloud fusion have yielded many high-accuracy results, most of these studies rely on selected GCPs or check points for evaluation. While the use of GCPs is a common practice for evaluating registration accuracy, relying solely on a few discrete points may not fully capture spatial variations. Therefore, in this study, we also evaluate the UAV–UAV coordinate transformation results by incorporating multiple verification zones—such as rooftops, pavements, tree canopies, and grasslands—to provide a more spatially representative assessment of accuracy. This approach will provide a more robust and credible assessment than relying solely on GCPs. On the other hand, unmanned surface vehicle (USV) technology has been widely used in recent years, including for depth measurements in ports, ponds, and reservoirs. This provides important insights into sediment accumulation and the inspection of underwater structures in these areas [25,26,27,28]. If USVs are combined with UAVs, the integration of the USV-UAV point cloud model can be used to construct a comprehensive aquatic environment, including both above-water and underwater terrains, which is crucial for the further understanding of sediment transportation and ecological environments. While some studies have addressed UAV and USV data integration, research specifically dealing with the registration of USV–UAV point cloud data for this purpose is still limited. Due to the limited research on UAV-UAV and USV-UAV point cloud fusion, this study aims to address two key issues. First, the results of the USV and UAV point cloud registration are investigated to construct a comprehensive terrain model of the aquatic environment, which will be followed by accuracy evaluation. Second, we explore the outcomes of fusing point clouds from two UAV-SfM (structure from motion) datasets to overcome the current limitations of low-cost drones in terms of the flight range and the number of images processed by photogrammetry software. The entire integration of the USV-UAV and UAV-UAV point cloud models was conducted via 4-parameter, 6-parameter, and 7-parameter coordinate transformation methods (CT methods), which are all essential for precise geodetic coordinate conversions and aligning different geospatial datasets. All the point cloud fusion results were uploaded to the Pointbox website, allowing anyone to easily access and view the research findings.

## 2. Methods

### 2.1. UAVs and USVs

This study uses SfM-based photogrammetry principles to construct point cloud models from UAV data. SfM photogrammetry is a technique used to reconstruct 3D scenes from a collection of 2D images captured from different viewpoints. The point cloud models generated from the UAV in this study were produced via Pix4Dmapper 4.8.4 software. SfM photogrammetry consists of a series of computer vision and photogrammetric methods for reconstructing scene structure and camera motion from overlapping images [28]. The SfM-based photogrammetry approach was employed to construct point cloud models from UAV imagery. The models were generated using Pix4Dmapper software, which applies SfM and multi-view stereo (MVS) algorithms to reconstruct 3D surfaces from overlapping 2D images. SfM integrates computer vision and photogrammetric methods to recover both scene structure and camera motion from image centralization [29]. The SfM process typically includes key steps such as feature extraction and matching, camera pose estimation, bundle adjustment, and 3D reconstruction. In the feature extraction and matching stage, key feature points are detected from UAV images, usually at distinct textures or edges (e.g., building corners or road markings). The scale-invariant feature transform (SIFT) algorithm [30] is used to extract and match corresponding feature points across multiple images. In the camera pose estimation step, the geometric relationships of collinearity and epipolar geometry are used to compute the UAV camera positions, motion directions, and intrinsic parameters, thereby constructing a sparse 3D point cloud structure. To minimize projection errors caused by image misalignment or overlap inconsistencies, bundle adjustment is performed in Pix4Dmapper to refine image orientation and improve reconstruction accuracy. The objective function of the bundle adjustment can be expressed as follows [31]:

(1) $$\sum_{i=1}^{n}{\left\|p_{i}-P_{i}(X_{i},R_{i},T_{i})\right\|}^{2}$$

where $p_{i}$ is the observed image point, $P_{i}$ is the projected model from the 3D coordinates $X_{i}$, $R_{i}$ is the rotation matrix, and $T_{i}$ is the translation vector. Finally, the MVS algorithm [32] generates a dense point cloud and reconstructs the 3D surface by estimating depth maps from feature correspondences across multiple overlapping images. These algorithms are well established; therefore, only a concise description is provided here, as the main focus of this study lies in the registration of UAV and USV point clouds, rather than the image-reconstruction procedure.

The purpose of USV depth sounding is to calculate the water depth via the speed and time difference of the sound waves traveling through water. The depth sounding technology adopted in this study is the multibeam echo sounding system. When a USV equipped with a multibeam echo sounder travels on the water surface, it emits a sound wave toward the bottom. When the sound wave hits the bottom, it reflects back. Once the receiver receives the reflected sound wave, it can calculate the travel time of the sound wave, thereby determining the water depth h, as shown below [33]:

(2) $$h=\frac{1}{2}(v\times t)+k+d$$

where v is the speed of sound in water, t is the round-trip travel time of the sound wave, k is a constant correction factor used to compensate for the system time delay between signal transmission and reception, and d is the draft of the vessel, representing the vertical distance between the water surface and the transducer position. The inclusion of k and d ensures that the calculated water depth reflects the true distance from the water surface to the seabed. The system used in this study was a NORBIT iWBMS multibeam echo sounder (NORBIT Subsea, Trondheim, Norway), operating at a frequency range of 200–700 kHz with a depth accuracy of approximately ±5 cm. In addition, the USV system (NORBIT iWBMS) included a built-in time synchronization function between the GNSS receiver and the multibeam sonar sensors, ensuring that all depth measurements were temporally aligned with the GNSS positioning data. Combined with the UAV GNSS, this setup provided a consistent spatio-temporal reference framework for both datasets. Because the UAV and USV surveys were conducted on the same day under calm conditions and within a small study area, additional extrinsic calibration was not required.

### 2.2. Coordinate Transformation

The CT strategies for this study include 4-parameter, 6-parameter, and 7-parameter strategies. The 4-parameter CT is used for simple adjustments, such as translating and rotating maps with a uniform scale [34,35]. The 6-parameter CT handles cases where differential scaling and rotation are needed [35,36]. The 7-parameter Helmert transformation is a commonly used 3D coordinate transformation model that consists of three translation parameters, three rotation parameters, and one uniform scale factor [37,38]. It is widely applied in the registration of point cloud data obtained from different platforms, such as UAVs and USVs, to achieve spatial alignment under a single global scale. In contrast, the 4-parameter and 6-parameter transformations are typically applied to 2D datasets, where the parameters include translations, rotation, and (in the case of the 4-parameter model) a scale factor. Although the 7-parameter model assumes uniform scaling, future studies could explore extending this model by introducing additional scale factors to form 8- or 9-parameter transformations for anisotropic scaling and enhanced registration flexibility. The 4-parameter CT model can be expressed as follows [39]:

(3) $$\left\{\begin{array}{c} X=ax-by+c\\ Y=bx-ay+d \end{array}\right.$$

where (x, y) are the original coordinates, and (X, Y) are the transformed coordinates. It includes the translation parameters (c and d) and the rotation parameters (a and b). The six-parameter CT model can be expressed as follows [39]:

(4) $$\left\{\begin{array}{c} X=ax+by+c\\ Y=dx+ey+f \end{array}\right.$$

where (x, y) are the original coordinates, and (X, Y) are the transformed coordinates. It includes the translation parameters (c and f), the rotation parameters and scale adjustments (a, b, d, and e). The 7-parameter CT model between any two Cartesian systems can be written as follows [40]:

(5) $$\left[\begin{array}{c} X_{G}\\ Y_{G}\\ Z_{G} \end{array}\right]=\left(1+dm\right)\left[\begin{array}{ccc} 1 & \gamma & -\beta \\ -\gamma & 1 & \alpha \\ \beta & -\alpha & 1 \end{array}\right]\left[\begin{array}{c} X_{L}\\ Y_{L}\\ Z_{L} \end{array}\right]+\left[\begin{array}{c} T_{X}\\ T_{Y}\\ T_{Z} \end{array}\right]$$

where ($X_{L}$, $Y_{L}$, $Z_{L}$) are the original coordinates and ($X_{G}$, $Y_{G}$, $Z_{G}$) are the transformed coordinates;${\;T}_{X}$, $T_{Y}$, and $T_{Z}$ are the translation parameters; α, β, and γ are the rotation parameters; and dm is the parameter of scale correction. In this study, CT calculations involving four parameters, six parameters, or seven parameters are all determined through a least squares adjustment.

In Equations (3)–(5), each parameter represents a distinct geometric element of the transformation. In the 4-parameter and 6-parameter models, the coefficients (a, b, d, e) describe the combined effects of rotation and scale in the horizontal plane, while the remaining terms (c, d, f) represent translations along the X and Y directions. For the 7-parameter Helmert transformation (Equation (5)), $T_{X}$, $T_{Y}$, and $T_{Z}$ denote translations, α, β, and γ are the rotation angles about the X-, Y-, and Z-axes, and dm is the uniform scale factor. The rotations follow the right-hand rule, where positive angles correspond to counter-clockwise rotation about each axis. The transformation is applied in the X–Y–Z order to convert coordinates from the local system ($X_{L}$, $Y_{L}$, $Z_{L}$) to the global system ($X_{G}$, $Y_{G}$, $Z_{G}$). This convention is consistent with the standard geodetic Helmert model commonly used in 3D coordinate conversion.

## 3. Study Area and Data

**Figure 1:**
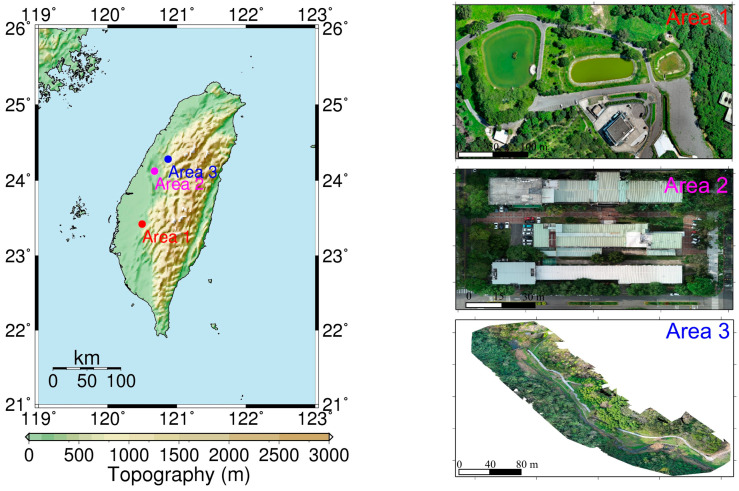
**Left** image: Taiwan topography and the locations of study areas 1–3. **Right**: Aerial orthophotos of study areas 1–3.

This paper includes three study areas. Their locations and aerial images are shown in Figure 1. Study area 1 is located in the Cien retention basin area in Zhongpu Township, Chiayi County. The area comprises three ponds, with this study using the central pond as a case example for the registration of USV and UAV point cloud data. The USV was employed to survey the bathymetry of the pond bottom, whereas the UAV was used to survey the terrain around this pond. Study area 2 is located at the buildings of the Department of Soil and Water Conservation (SWC) of National Chung Hsing University (NCHU), Taichung City, and study area 3 is located at the Sijiaolin Stream within Dongshi Forestry Culture Park in Taichung City. There are several soil and water conservation structures in the Sijiaolin Stream. We test the registration results of the USV-derived and UAV-derived point cloud models in the study area 1 and those of the UAV-derived point cloud models in the study areas 2 and 3.

For each study area, control points were selected based on geometric distinctiveness, clear visibility in both UAV and USV datasets, and adequate spatial coverage. Points were arranged as uniformly as possible within the overlap or boundary zones to minimize spatial bias, while collinear configurations were avoided to maintain geometric stability. All control points corresponded to sharp and easily identifiable terrain features—such as embankment corners, building edges, and spillway intersections—ensuring repeatable and reliable registration across datasets. The control points were selected as comprehensively as possible within each study area, considering the geometric and visibility constraints of both UAV and USV datasets. Although some regions offered limited distinctive features due to vegetation or water-surface reflections, the selected points provided sufficient spatial coverage and geometric stability for reliable registration.

**Figure 2:**
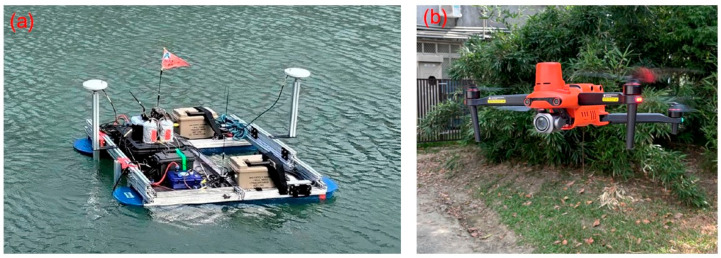
(**a**) USV system of Chen Kai Technology. (**b**) AUTEL EVO II.

**Figure 3:**
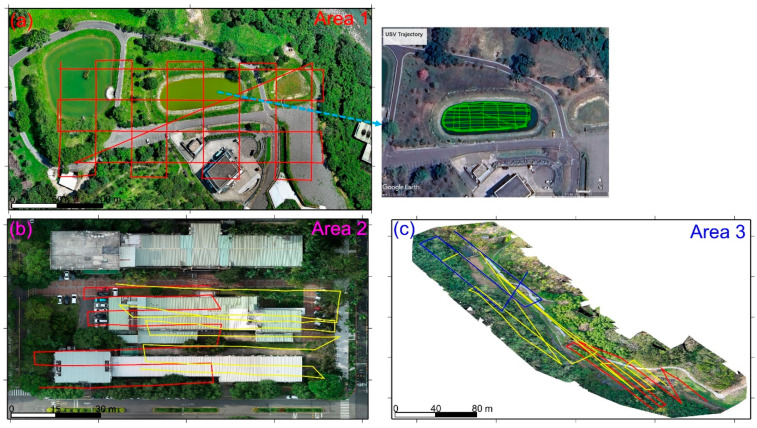
(**a**) UAV trajectories (red lines) of study area 1, with an inset on the right showing the USV trajectories (green lines). (**b**) UAV trajectories of study area 2, with red and yellow dots representing Models 2-1 and 2-2, respectively. (**c**) UAV trajectories of study area 3, with red, yellow, and blue lines representing Models 3-1, 3-2, and 3-3, respectively.

The USV system used in study area 1 is the NORBIT iWBMS multibeam sonar system [41] mounted in an unmanned vehicle from Chen Kai Technology (Figure 2a). Additionally, the drone used in study areas 1–3 is the AUTEL EVO II, as shown in Figure 2b. The trajectories of the USV and UAV missions in study areas 1–3 are shown in Figure 3. In Figure 3a, the red dots and green dots represent the UAV and USV mission trajectories, respectively. The photos over study area 1 were taken on 4 July 2023, at an altitude of 80 m, with a forward overlap of 70% and a side overlap of 60%, totaling 151 photos. The USV survey in study area 1 was also conducted on 4 July 2023, yielding a total of 502,521 point cloud data points. In Figure 3b, the trajectories with red and yellow dots represent Models 2-1 and 2-2, respectively. The aerial photography dates for the two models were 23 October 2023, and 24 October 2023. The flight altitude was 30 m, with a forward overlap of approximately 70% and a side overlap of approximately 60%. The number of aerial photos taken was 53 for Model 2-1 and 99 for Model 2-2. In Figure 3c, the trajectories with red, yellow, and blue dots represent Models 3-1, 3-2, and 3=3, respectively. The UAV aerial photography date for study area 3 was 4 March 2024. The flight altitude was 100 m, with a forward overlap of approximately 80%, and a side overlap of approximately 70%. The number of aerial photos taken for Models 3-1 to 3-3 was 43, 106, and 55, respectively. Although there are three point cloud models in study area 3, this paper analyzes only the registration results of Models 3-1 and 3-2. All of the registrations of the USV-derived and UAV-derived point cloud models in the three study areas are implemented with 4-parameter, 6-parameter, and 7-parameter CT methods.

## 4. Results and Discussion

### 4.1. The Results for Study Area 1: USV–UAV Point Cloud Registration

**Figure 4:**
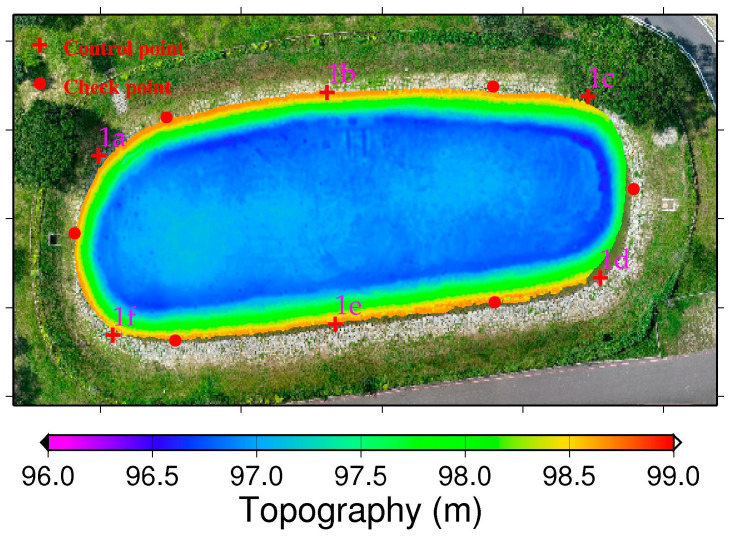
The topography of the farm pond measured by the USV, with red cross points (1a–1f) and red circles indicating control and check points used for UAV/USV point cloud fusion.

Since USV and UAV technologies are used for measuring underwater and ground terrain, respectively, their point cloud models do not overlap. However, we selected 6 control points and 6 check points in areas where the two point cloud models are in close proximity, and where features are distinct, such as embankment gaps and spillway corners. In Figure 4, the red cross points and red circles indicate the control and check points, respectively, with the control and check points being alternately distributed. All the control points are utilized with the 4-parameter, 6-parameter, and 7-parameter CT methods to estimate the unknown parameter values using the least squares approach. Then, the horizontal coordinate of the USV-derived point cloud model is transformed to a new horizontal coordinate consistent with that of the UAV. For the elevation coordinates, since the 4-parameter and 6-parameter CTs are 2D transformations, we use a single control point at the spillway corner to adjust the elevation coordinates of the USV to those of the UAV.

**Figure 5:**
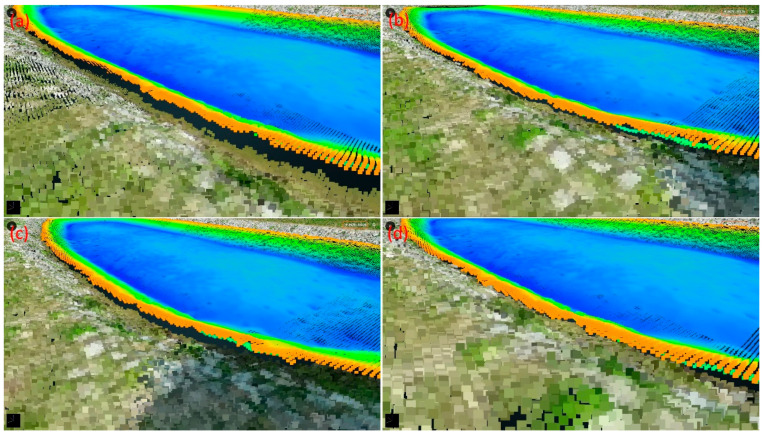
The partial fusion results of USV-UAV point clouds in study area 1: (**a**) without the CT method, (**b**) with the 4-parameter CT method, (**c**) with the 6-parameter CT method, and (**d**) with the 7-parameter CT method. The entire point cloud fusion results for (**a**–**d**) can be found at the following four links (accessed on 30 August 2024): https://www.pointbox.xyz/clouds/66b32be2817a42b2255567e2, https://www.pointbox.xyz/clouds/66b3257c817a4296c65567da, https://www.pointbox.xyz/clouds/66b328eb817a4256e35567de, https://www.pointbox.xyz/clouds/66b32b84817a42e34f5567e0.

**Figure 6:**
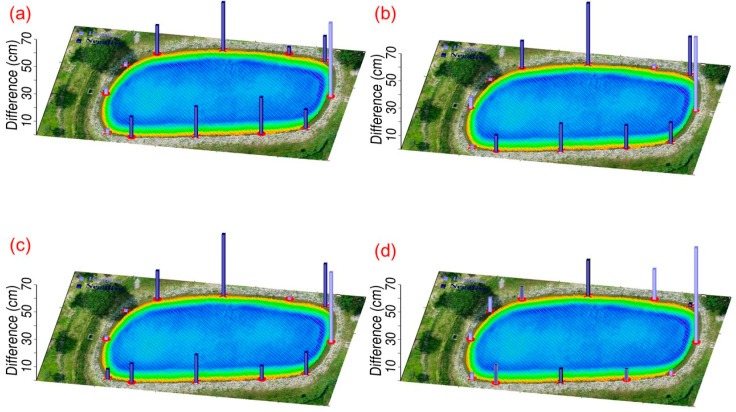
The height differences (vertical bars) between UAV and USV point clouds at the control and check points in study area 1: (**a**) without the CT method, (**b**) with the 4-parameter CT method, (**c**) with the 6-parameter CT method, and (**d**) with the 7-parameter CT method. The light purple and dark purple bars indicate positive and negative values, respectively.

Figure 5 shows a portion of the fusion results of the USV–UAV point clouds in study Area 1. The Pointbox links for all the point cloud fusion results are provided in the caption of Figure 5. The spatial resolutions of the USV and UAV point cloud models shown in the links are 10 cm and 3 cm, respectively. In Figure 5, without CT, there are noticeable gaps between the UAV-based and USV-based point clouds (Figure 5a). However, after applying CT, no matter whether the 4-parameter, 6-parameter, or 7-parameter CT methods are used, the gaps are reduced (Figure 5b–d). Table 1 shows the coordinate differences at each control point before and after applying CT. Since the 4-parameter and 6-parameter CT methods do not account for the Z-direction (elevation), the standard deviations for these methods, as presented in Table 1, are calculated solely in the horizontal plane. In contrast, the standard deviation for the 7-parameter CT method is computed in 3D space. Furthermore, Table 1 reveals that the standard deviations obtained using the 4-parameter, 6-parameter, and 7-parameter CT methods range from 31 to 34 cm, demonstrating decimeter-level accuracy. Since the Z-direction component in the 4-parameter and 6-parameter CT methods is treated as a single translation, we further compared their performance with the 7-parameter CT method in terms of Z-direction coordinate transformation. To do so, we analyzed the elevation differences of all control and check points before and after applying CT. The results are presented in Figure 6. The standard deviations of the differences for the cases without the CT method, with the 4-parameter CT method, with the 6-parameter CT method, and with the 7-parameter CT method—comprising both control and check points—are 17.5 cm, 16.3 cm, 12.9 cm, and 14.6 cm, respectively. These statistics indicate that the application of the CT method for point cloud fusion results in a reduction in standard deviation, with the 6-parameter method yielding the best results. This can be attributed to the fact that both the control and check points are located at the water surface interface, ensuring elevation consistency. As a result, the advantages of the 3D 7-parameter transformation are less pronounced compared to those of the 2D 6-parameter transformation. Additionally, the standard deviation results show that although the use of CT methods led to improved outcomes compared to non-CT methods, the improvement was not substantial. The primary reason for this is that, due to the lack of overlapping point clouds from the USV and UAV missions, control and check points can only be selected from the boundary areas, which may introduce selection errors.

Overall, although the point cloud fusion results of the USV–UAV integration achieved only decimeter-level accuracy, the results still provide valuable insights into local sediment transport and the ecological environment in retention basins.

### 4.2. Results for Study Area 2: UAV/UAV Point Cloud Fusion

**Figure 7:**
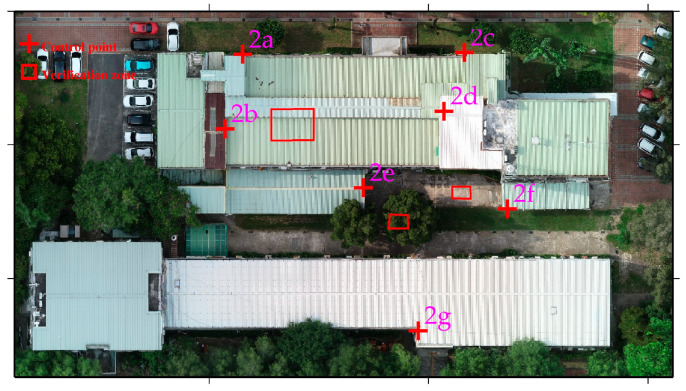
Aerial orthophoto of study area 2, with the red cross points (2a–2g) and frames indicating the control points and verification zones used for UAV point cloud integration. The verification zones include rooftops, tree canopies, and pavements.

**Figure 8:**
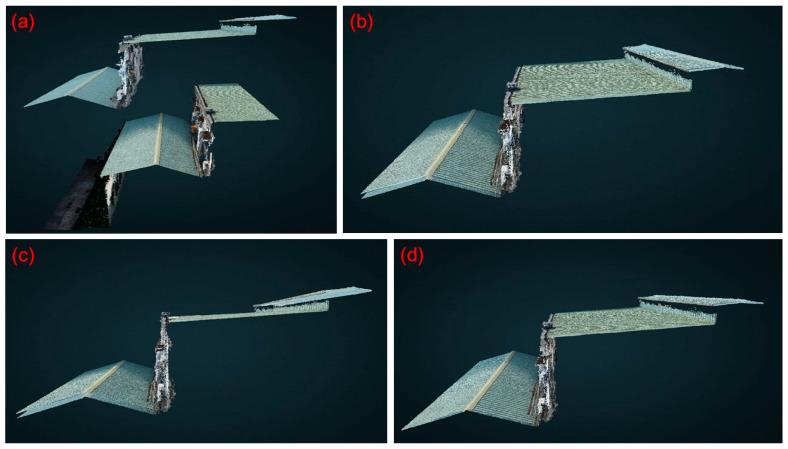
The partial integration results of UAV-based point cloud Models 2-1 and 2-2 in study area 2: (**a**) without the CT method, (**b**) with the 4-parameter CT method, (**c**) with the 6-parameter CT method, and (**d**) with the 7-parameter CT method. The entire point cloud integration results for (**a**–**d**) can be found at the following four links (accessed on 30 August 2024): https://www.pointbox.xyz/clouds/66b37dfb817a428b49556804, https://www.pointbox.xyz/clouds/66b330a2817a42933b5567e6, https://www.pointbox.xyz/clouds/66b33288817a42ba785567e8, https://www.pointbox.xyz/clouds/66b33446817a425bbb5567ea.

**Figure 9:**
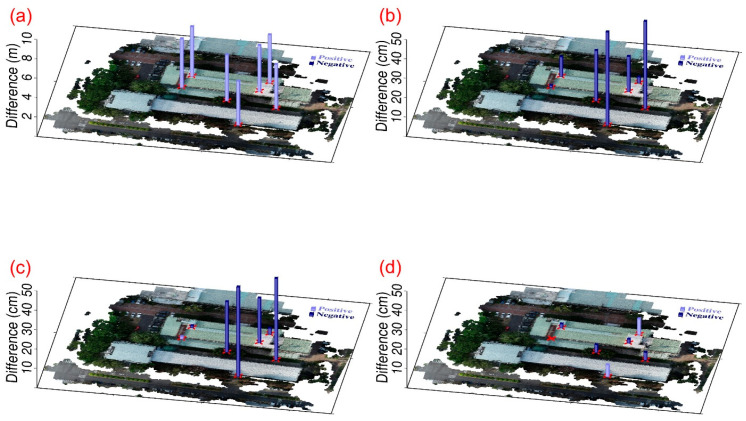
The elevation differences (vertical bars) between point cloud Models 2-1 and 2-2 at the control points in study area 2: (**a**) without the CT method, (**b**) with the 4-parameter CT method, (**c**) with the 6-parameter CT method, and (**d**) with the 7-parameter CT method. The light purple and dark purple bars indicate positive and negative values, respectively.

Study area 2 was located at the buildings of the Department of SWC on the NCHU campus. We tested the 4-parameter, 6-parameter, and 7-parameter CT methods for the point cloud registration of Models 2-1 and 2-2. Seven control points were selected at the corners of the building for easy identification, and their distribution is shown in Figure 7. In addition, we did not select specific check points in this area but instead chose three verification zones, which are the red frames shown in Figure 7. The verification zones include rooftops, tree canopies, and pavements. The 4-parameter, 6-parameter, and 7-parameter CT methods use the 7 control points to solve for unknown parameter values. We then transform the Model 2-2 coordinate system to the Model 2-1 coordinate system. Since the 4-parameter and 6-parameter CTs are both 2D transformations, we use one control point at the rooftop corner to adjust the Model 2-2 elevation. Figure 8 shows a portion of the registration results (rooftop) of point cloud Models 2-1 and 2-2 in study Area 2. Without the use of the CT method, the difference between Models 2-1 and 2-2 is quite pronounced (Figure 8a). However, when the 4-parameter and 6-parameter CT methods are used, the gap between the two models is significantly reduced (Figure 8b,c), although some minor differences are still evident. With the 7-parameter method, the two models are almost merged into one. The Pointbox links for the complete point cloud registration results shown in Figure 8 are summarized in the figure caption. The spatial resolution is 3 cm. Table 2 presents the coordinate differences at each control point before and after applying the CT methods. The same as Table 1, the standard deviations for 4-parameter and 6-parameter CT methods are calculated solely in the horizontal plane, and 7-parameter CT method is computed in 3D space. Furthermore, Table 2 reveals that the standard deviations obtained using the 4-parameter, 6-parameter, and 7-parameter CT methods range from 4 to 6 cm, demonstrating centimeter-level accuracy. Since the Z-direction component in the 4-parameter and 6-parameter CT methods is treated as a single translation, we further compared their performance with the 7-parameter CT method in terms of Z-direction coordinate transformation, as shown in Figure 9. Figure 9 shows that without the use of the CT method, the difference between Models 2-1 and 2-2 at the control points reaches several meters. After applying the 4-parameter or 6-parameter CT methods, the difference decreases to several tens of centimeters, and with the use of the 7-parameter CT method, it further decreases to several centimeters.

To further comprehensively evaluate the CT results for study area 2, we assessed the accuracy using the verification zones. First, we used GMT 6.5 software [42] to grid the elevations of the verification zones (with a grid spacing of 10 cm), including the zones in Models 2-1 and 2-2, both before and after applying CT. We then calculated and statistically analyzed the elevation differences at each grid point and compute the correlation coefficients. Table 3 presents the statistics of the elevation differences between Model 2-1 and Model 2-2 across the various verification zones. The table shows that, without the use of the CT method, the mean elevation difference in any verification zone reaches several meters. In terms of standard deviation, due to the flat terrain of the pavement, only the verification zone corresponding to the pavement produces a relatively good result, with a standard deviation of 4.9 cm, whereas the standard deviations for the rooftop and tree canopy verification zones are in the meter range. After applying the 4-parameter or 6-parameter CT method, both the mean value and standard deviation in the three verification zones are significantly reduced, with the mean value ranging from approximately −8 to −37 cm, and the standard deviation decreasing to around 1 to 37 cm. After the 7-parameter CT method is applied, the mean values and standard deviations in the verification zones—rooftop and pavements—further decrease. The mean values are between −3.8 and −6.5 cm, and the standard deviations range from 0.6 to 0.9 cm, indicating that the 7-parameter method provides highly ideal point cloud integration results for these zones. However, for the verification zone of the tree canopy, the results are comparable to those obtained with the 4-parameter or 6-parameter methods, with average values and standard deviations of approximately 16 and 36 cm, respectively. This is likely because tree canopies are areas with very high terrain roughness; even minor displacements in planar coordinates can result in significant elevation differences. Thus, even with the 7-parameter CT method, the accuracy cannot be reduced to the centimeter level at the verification zone of the tree canopy.

Table 4 shows the correlation coefficients between Model 2-1 and Model 2-2 in each verification zone under different CT methods. Table 4 shows that the use of the CT method yields the best results in the verification zone of the rooftop. The correlation coefficient improves from 0.44 without CT to over 0.9, and with the 7-parameter CT method, it even reaches 0.99. The same results are observed in the verification zone of the pavement. However, in the verification zone of the tree canopy, the results with, and without, the CT method are similar, ranging from 0.78 to 0.83, due to the very high terrain roughness of the tree canopy. Overall, in the study of point cloud integration in study area 2, the results of the 7-parameter CT method are significantly better than those of the 4-parameter and 6-parameter CT methods. Although the 7-parameter CT method performs best, Table 3 shows that the 4-parameter and 6-parameter CT methods can still achieve centimeter-level accuracy or even better on rooftops and pavements. This is likely due to the even distribution of control points in this study area.

### 4.3. Results for Study Area 3: UAV/UAV Point Cloud Fusion

**Figure 10:**
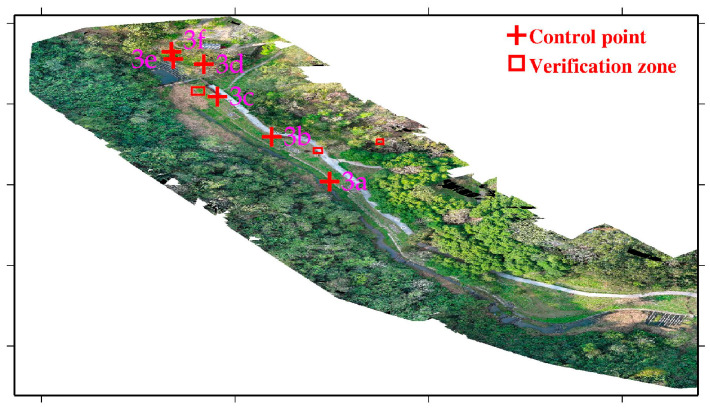
Aerial orthophoto of study area 3, with the red cross points (3a–3f) and frames indicating the control points and verification zones used for UAV point cloud fusion. The verification zones include the tree canopy, pavement, and grassland.

**Figure 11:**
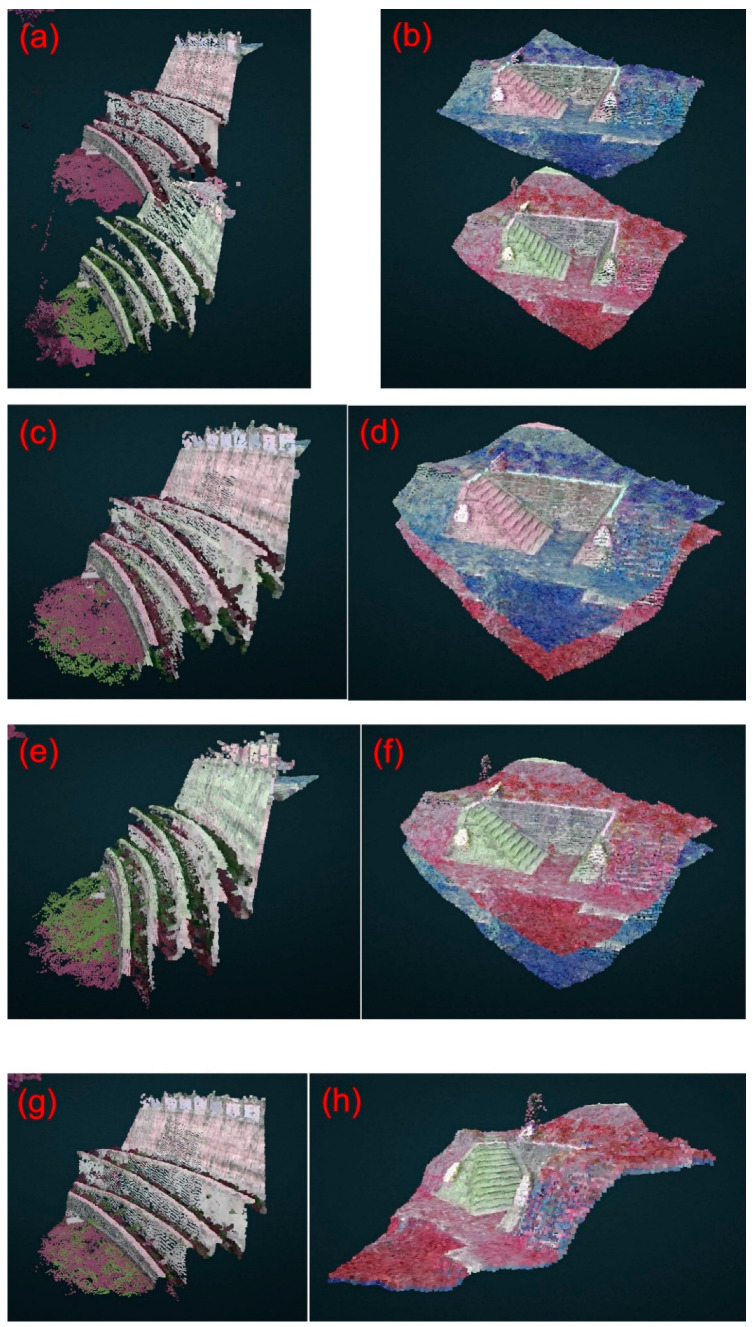
The partial integration results of point cloud Models 3-1 and 3-2 in study area 3: (**a**,**b**) without the CT method, (**c**,**d**) with the 4-parameter CT method, (**e**,**f**) with the 6-parameter CT method, and (**g**,**h**) with the 7-parameter CT method. The entire point cloud fusion results for (**a**–**d**) can be found at the following four links (accessed on 30 August 2024): https://www.pointbox.xyz/clouds/66b37adb817a4275f75567fc, https://www.pointbox.xyz/clouds/66b37cd3817a42f55d5567fe, https://www.pointbox.xyz/clouds/66b37dcc817a428987556800, https://www.pointbox.xyz/clouds/66b37de0817a423873556802.

Study area 3 was located at the Sijiaolin stream within the Dongshi forestry culture park. We tested the 4-parameter, 6-parameter, and 7-parameter CT methods for the point cloud integration of Model 3-1 and Model 3-2. Six control points were selected at the walkways, which are easy to identify. The distribution of all control points is shown in Figure 10. We additionally used three verification zones (a grid spacing of 10 cm), which are the red frames shown in Figure 10, to evaluate the point cloud integration results. The verification zones include tree canopies, pavements, and grasslands. The 4-parameter, 6-parameter, and 7-parameter CT methods use the 6 control points to solve for unknown parameter values. Then, we transform the Model 3-2 coordinate system to the Model 3-1 coordinate system. Since both the 4-parameter and the 6-parameter CT are 2D transformations, we used one stable control point to adjust the elevation of Model 3-2. Figure 11 shows a portion of the integration results of Models 3-1 and 3-2. Figure 11a,c,e,g show the structures of the fishways, whereas Figure 11b,d,f,h depict the structures of the wildlife passages. Without the use of the CT method, the difference between Models 3-1 and 3-2 is quite pronounced (Figure 11a,b). However, when the 4-parameter and 6-parameter CT methods are used, the gap between the two models is significantly reduced (Figure 11c–f), although some minor differences are still evident. With the 7-parameter method, the two models are almost merged into one (Figure 11g,h). In addition, the estimated scale factors obtained from the 7-parameter CT were close to unity (within ±0.001), confirming that the UAV and USV point clouds were consistent in physical scale after alignment to the same reference frame defined by the USV GNSS positioning. Since the area of the Sijiaolin stream is mostly forested, to visualize the integration results of Models 3-1 and 3-2 in Figure 11, we have represented the forest and grassy areas of Models 3-1 and 3-2 in red and blue, respectively. The Pointbox links of the entire point cloud integration results are summarized in the caption of Figure 11. The spatial resolution of the two UAV point cloud models shown in the Pointbox links is 3 cm.

**Figure 12:**
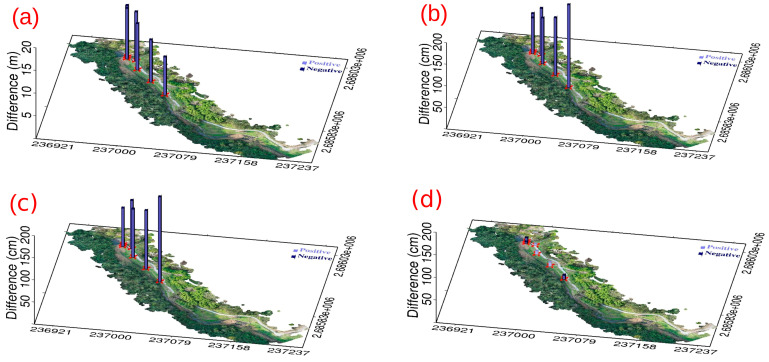
The elevation differences (vertical bars) between point cloud Models 3-1 and 3-2 at the control points in study area 3: (**a**) without the CT method, (**b**) with the 4-parameter CT method, (**c**) with the 6-parameter CT method, and (**d**) with the 7-parameter CT method. The light purple and dark purple bars indicate positive and negative values, respectively.

Table 5 presents the coordinate differences at each control point before and after applying the CT methods. The same as Table 1 and Table 2, the standard deviations for 4-parameter and 6-parameter CT methods are calculated solely in the horizontal plane, and 7-parameter CT method is computed in 3D space. Table 5 reveals that the standard deviations obtained using the 4-parameter, 6-parameter, and 7-parameter CT methods range from 5 to 7 cm, demonstrating centimeter-level accuracy. Since the Z-direction component in the 4-parameter and 6-parameter CT methods is treated as a single translation, we further compared their performance with the 7-parameter CT method in terms of Z-direction coordinate transformation, as shown in Figure 12. Figure 12 shows that without the use of the CT method, the difference at the control points between Model 3-1 and Model 3-2 reaches 10–20 m. After applying the 4-parameter or 6-parameter CT methods, the difference decreases to 1–2 m, and with the use of the 7-parameter CT method, it further decreases to several centimeters. Table 6 presents the statistics of the elevation differences between Model 3-1 and Model 3-2 in different verification zones. The Table shows that without CT, both the mean values and standard deviations for the three verification zones are the largest. After the 4-parameter or 6-parameter CT methods were applied, both the mean values and standard deviations decreased. Among these zones, the reductions in the verification zones of the pavement and grassland are more significant than those in the verification zone of the tree canopy. After the 7-parameter CT method was used, the mean value was similar to that obtained with the 4-parameter and 6-parameter CT methods, but the standard deviation was further reduced, reaching 33.9 cm, 11.8 cm, and 4.3 cm for the verification zones of the tree canopy, pavement, and grassland, respectively. Compared with the verification zones of the pavement and grassland, the result for the verification zone of the tree canopy is slightly worse because of the very high terrain roughness.

Table 7 shows the correlation coefficients between Model 3-1 and Model 3-2 in each verification zone under different CT methods. Without the CT method, the correlation coefficient between the two models in the verification zone of the tree canopy is relatively low, at only 0.79, whereas in the other two verification zones, it exceeds 0.9. After the CT method is applied, the correlation coefficients between the two models in all the verification zones significantly increase. In the verification zone of the canopy, the 7-parameter method yields the best correlation coefficient, reaching 0.97. In the other verification zones, all three CT methods yielded consistent correlation coefficients of 0.99. Overall, in the study of point cloud integration in study area 3, the results of the 7-parameter CT method are significantly better than those of the 4-parameter and 6-parameter CT methods. Compared to study area 2, the results of the 4-parameter and 6-parameter CT methods are noticeably worse than those of the 7-parameter CT method. This is likely due to the less even distribution of control points in this area.

## 5. Conclusions

This study investigates the integration of point cloud data from UAVs and USVs to generate comprehensive terrain models in aquatic environments, and from UAVs to develop continuous terrain models in urban and stream areas. The research includes three study sites: a retention basin in Chiayi County (study Area 1), the Department of SWC at NCHU (study Area 2), and the Sijiaolin stream (study Area 3). Point cloud models were generated using UAV and USV technologies, followed by integration of the USV-UAV and UAV-UAV data using 4-parameter, 6-parameter, and 7-parameter CT methods. These methods were employed to align disparate datasets and produce accurate geospatial models.

The results of the USV-UAV integration demonstrate that CT methods effectively reduce discrepancies between the USV and UAV point clouds, achieving decimeter-level accuracy across all three CT methods (4-, 6-, and 7-parameter) in study area 1. Among these, the 6-parameter CT method delivered the best performance, with a standard deviation of 12.9 cm in elevation differences. However, the overall improvement in standard deviation remained modest, primarily due to the non-overlapping nature of the point clouds and potential errors in control point selection. These findings emphasize the importance of precise control point selection and highlight the inherent challenges in integrating heterogeneous point cloud datasets. Overall, this study provides valuable insights into sediment transport and ecological conditions in retention basins.

The USV–UAV integration benefited from the built-in GNSS–sonar time synchronization in the USV system, which ensured temporal consistency between the datasets. However, for larger-scale or highly dynamic environments, detailed sensor calibration and synchronization procedures remain essential to minimize systematic errors.

The UAV-UAV integration results show that, regardless of the CT method (4-, 6-, or 7-parameter), the difference in control points before and after transformation consistently achieved centimeter-level accuracy. When validation was performed using verification zones, significant improvements were observed in reducing elevation discrepancies and achieving better point cloud alignment, particularly in areas with less terrain roughness, such as pavements and rooftops. In study area 2, CT methods achieved an accuracy of 1 to 3 cm for rooftops, and less than 1 cm for pavements. In study area 3, the CT methods resulted in accuracies of 11 to 19 cm for pavements and 4 to 14 cm for grasslands. These findings align with previous research. However, in areas with more complex terrain, such as tree canopies, accuracy improvements were less pronounced, although the 7-parameter CT method still outperformed the others. In general, the 7-parameter CT method consistently provided superior results compared to the 4-parameter and 6-parameter methods, in both study area 2 and 3. Notably, the 4- and 6-parameter methods yielded results closer to the 7-parameter method in study area 2, while in study area 3, the 4- and 6-parameter methods performed significantly worse. This discrepancy is attributed to the more even distribution of control points in study area 2 compared to study area 3.

The key contributions of this study are as follows: In USV-UAV integration research, this paper is the first to explore the acquisition of both underwater and above-water terrain data using USV-UAV systems and to perform point cloud model registration. These findings provide a valuable reference for future efforts in integrating UAV and USV point cloud data, facilitating more accurate and comprehensive modeling of diverse terrains, particularly in aquatic and adjacent environments. In the UAV-UAV research, this study evaluates the accuracy of different CT methods for registering UAV-based point cloud models. Notably, replacing traditional check points with verification zones may provide a more representative and comprehensive assessment.

While numerous studies on TLS-TLS, TLS-UAV, and UAV-UAV point cloud integration (e.g., [1,2,3,4,5,6,7,23]) report high accuracy, many rely on commercial or custom software for data processing, which involves complex computational procedures. Additionally, point cloud fusion results are often confined to specific commercial software platforms, limiting their broader applicability and dissemination. In contrast, this paper employs widely used CT methods, offering a simpler, more accessible approach to merging point cloud models. The results demonstrate that this method achieves high accuracy while remaining straightforward. Furthermore, all point cloud fusion results from this study are available on the Pointbox website, a free platform for viewing point cloud models, facilitating easy access and sharing of the research findings.

## Figures and Tables

**Table 1:** Statistics of the coordinate differences at control points among the different CT methods (unit: cm) in study area 1.

CT Method	Control Point	X-Diff	Y-Diff	Z-Diff	Std. Dev.
4-parameter	1a	−8.0	−8.4	--	32.3
	1b	18.3	15.0	--	
	1c	8.8	−18.1	--	
	1d	−23.0	−31.6	--	
	1e	1.8	70.4	--	
	1f	2.1	−27.3	--	
6-parameter	1a	−16.0	−8.6	--	31.4
	1b	9.3	20.1	--	
	1c	4.6	−10.8	--	
	1d	−13.9	−32.1	--	
	1e	9.5	66.0	--	
	1f	6.5	−34.6	--	
7-parameter	1a	7.2	4.3	16.2	33.9
	1b	−11.7	−9.6	−36.9	
	1c	18.8	3.8	−2.0	
	1d	23.2	22.6	2.4	
	1e	−62.2	−33.2	−9.6	
	1f	24.6	12.0	3.6	

**Table 2:** Statistics of the coordinate differences at control points among the different CT methods (unit: cm) in study area 2.

CT Method	Control Point	X-Diff	Y-Diff	Z-Diff	Std. Dev.
4-parameter	2a	0.6	1.5	--	5.3
	2b	0.7	3.0	--	
	2c	10.0	−3.2	--	
	2d	−6.0	−4.5	--	
	2e	−2.8	3.0	--	
	2f	−3.1	3.7	--	
	2g	0.6	−3.5	--	
6-parameter	2a	−0.6	0.0	--	4.9
	2b	1.1	0.8	--	
	2c	7.5	−1.5	--	
	2d	−7.1	−3.4	--	
	2e	−2.0	2.6	--	
	2f	−2.7	5.2	--	
	2g	3.8	−3.7	--	
7-parameter	2a	−1.2	−0.1	1.1	5.6
	2b	−2.0	−1.3	0.4	
	2c	4.9	−4.3	11.4	
	2d	0.8	4.8	−1.0	
	2e	−2.9	0.7	−1.5	
	2f	−3.5	−0.1	−1.4	
	2g	3.9	0.3	2.3	

**Table 3:** Statistics of the elevation differences among the different verification zones and CT methods (unit: cm) in study area 2.

Verification Zone	CT Method	Mean	Max.	Min.	Std. Dev.
Rooftop	No	305.3	536.7	−57.9	157.6
	4-parameter	−8.5	−1.7	−14.5	2.5
	6-parameter	−9.5	−1.7	−15.2	2.7
	7-parameter	−3.8	−0.2	−6.9	0.9
Tree canopy	No	986.1	1263.6	238.4	287.5
	4-parameter	−18.4	161.2	−171.0	37.5
	6-parameter	−16.4	178.0	−144.2	35.4
	7-parameter	16.0	150.2	−107.0	36.7
Pavement	No	471.0	481.9	460.7	4.9
	4-parameter	−36.4	−34.0	−38.9	0.9
	6-parameter	−37.3	−34.0	−40.0	0.9
	7-parameter	−6.5	−4.9	−8.1	0.6

**Table 4:** The correlation coefficients of Models 2-1 and 2-2 at different verification zones using different CT methods.

	No	4-Parameter	6-Parameter	7-Parameter
Rooftop	0.44	0.92	0.91	0.99
Tree canopy	0.82	0.79	0.83	0.78
Pavement	−0.88	0.71	0.63	0.88

**Table 5:** Statistics of the coordinate differences at control points among the different CT methods (unit: cm) in study area 3.

CT Method	Control Point	X-Diff	Y-Diff	Z-Diff	Std. Dev.
4-parameter	3a	1.1	0.9	--	7.0
	3b	−6.5	3.8	--	
	3c	7.2	−8.8	--	
	3d	4.2	−6.3	--	
	3e	−4.9	−0.8	--	
	3f	−1.1	11.2	--	
6-parameter	3a	4.1	4.6	--	5.3
	3b	−2.2	−4.1	--	
	3c	−8.4	−5.4	--	
	3d	0.3	−1.4	--	
	3e	2.8	−0.4	--	
	3f	3.4	6.6	--	
7-parameter	3a	−0.7	8.0	−4.8	7.2
	3b	2.0	−7.9	3.6	
	3c	−3.4	−7.5	4.3	
	3d	4.9	−2.7	2.9	
	3e	1.7	7.3	−3.2	
	3f	−4.5	2.9	−5.2	

**Table 6:** Statistics of the elevation differences among the different verification zones and CT methods (unit: cm) in study area 3.

Verification Zone	CT Method	Mean	Max.	Min.	Std. Dev.
Tree canopy	No	−468.8	−241.3	−682.9	83.9
	4-parameter	−217.6	−36.0	−404.2	77.6
	6-parameter	−220.9	−62.1	−396.1	69.5
	7-parameter	−72.7	31.5	−169.0	33.9
Pavement	No	−124.1	−370.3	115.1	101.7
	4-parameter	−2.7	−142.0	103.6	19.9
	6-parameter	−2.0	−135.2	88.2	20.3
	7-parameter	−2.4	−123.0	52.6	11.8
Grassland	No	−260.3	−191.6	−358.1	27.2
	4-parameter	−65.6	−30.6	−121.1	14.8
	6-parameter	−65.6	−29.6	−115.4	14.9
	7-parameter	−19.5	2.0	−83.5	4.3

**Table 7:** The correlation coefficients of Models 3-1 and 3-2 at different verification zones using different CT methods.

	No	4-Parameter	6-Parameter	7-Parameter
Tree canopy	0.79	0.85	0.87	0.97
Pavement	0.91	0.99	0.99	0.99
Grassland	0.97	0.99	0.99	0.99

## Data Availability

The UAV and USV data were generated and processed by groups from National Chung Hsing University and National Cheng Kung University, respectively. The data are available from the corresponding author upon reasonable request.
